# Mechanisms of resistance and sensitivity to anti-HER2 therapies in HER2+ breast cancer

**DOI:** 10.18632/oncotarget.7043

**Published:** 2016-01-27

**Authors:** Debora de Melo Gagliato, Denis Leonardo Fontes Jardim, Mario Sergio Pereira Marchesi, Gabriel N. Hortobagyi

**Affiliations:** ^1^ Centro de Oncologia do Paraná, Curitiba SP, Brazil; ^2^ Roche Pharmaceuticals, São Paulo SP, Brazil; ^3^ The University of Texas MD Anderson Cancer Center, Houston, TX, USA

**Keywords:** breast cancer, HER2 disease, trastuzumab, anti-HER2 therapy, resistance to treatment

## Abstract

Breast Cancer (BC) is a highly prevalent disease. A woman living in the United States has a 12.3% lifetime risk of being diagnosed with breast cancer [1]. It is the most common female cancer and the second most common cause of cancer death in women [2]. Of note, amplification or overexpression of Human Epidermal Receptor 2 (HER2) oncogene is present in approximately 18 to 20% of primary invasive breast cancers, and until personalized therapy became available for this specific BC subtype, the worst rates of Overall Survival (OS) and Recurrence-Free Survival (RFS) were observed in the HER2+ BC cohort, compared to all other types, including triple negative BC (TNBC) [3].

HER2 is a member of the epidermal growth factor receptor (EGFR) family. Other family members include EGFR or HER1, HER3 and HER4. HER2 can form heterodimers with any of the other three receptors, and is considered to be the preferred dimerization partner of the other HER or ErbB receptors [4]. Phosphorylation of tyrosine residues within the cytoplasmic domain is the result of receptor dimerization and culminates into initiation of a variety of signalling pathways involved in cellular proliferation, transcription, motility and apoptosis inhibition [5].

In addition to being an important prognostic factor in women diagnosed with BC, HER2 overexpression also identifies those patients who benefit from treatment with agents that target HER2, such as trastuzumab, pertuzumab, trastuzumab emtansine (T-DM1) and small molecules tyrosine kinase inhibitors of HER2 [6, 11, 127]. In fact, trastuzumab altered the natural history of patients diagnosed with HER2+ BC, both in early and metastatic disease setting, in a major way [8–10]. Nevertheless, there are many women that will eventually develop metastatic disease, despite being treated with anti-HER2 therapy in the early disease setting. Moreover, advanced tumors may reach a point where no anti-HER2 treatment will achieve disease control, including recently approved drugs, such as T-DM1.

This review paper will concentrate on major biological pathways that ultimately lead to resistance to anti-HER2 therapies in BC, summarizing their mechanisms. Strategies to overcome this resistance, and the rationale involved in each tactics to revert this scenario will be presented to the reader.

## INTRODUCTION: LANDMARK IMPROVEMENTS IN HER2+ DISEASE

Since the approval of trastuzumab in the adjuvant setting by the FDA in 2006, most patients with HER2+ early BC are doing very well with standard trastuzumab therapy added to chemotherapy. Planned Joint Analysis of OS from NSABP B-31 and NCCTG N9831, demonstrated that the addition of trastuzumab to a taxane and anthracycline chemotherapy backbone resulted in a durable improvement in survival, after a median time on study of approximately eight and a half years. [11]

In fact, patients nearly diagnosed with localized HER2+ BC have an excellent prognosis, even if treated with less toxic adjuvant systemic therapy, as recently demonstrated by Dana Farber investigators [10]. This nonrandomized prospective trial evaluated an adjuvant chemotherapy regimen consisting of weekly paclitaxel at 80mg/m^2^ and trastuzumab at 2 mg/kg for 12 weeks, followed by 9 months of trastuzumab in 406 women with HER2-positive, node-negative tumors ≤ 3 cm. Disease-free survival achieved an impressive result at 3 years of 98.7% (*P* < .0001), and the regimen was associated with great cardiac safety. [12]

In the metastatic setting, Slamon et al. [8] evaluated the addition of trastuzumab to chemotherapy among women diagnosed with metastatic HER2+ BC in the landmark trial that lead to trastuzumab approval in the metastatic setting. The authors found that the addition of trastuzumab to chemotherapy was associated with a longer time to disease progression, higher rate of objective response, and a longer survival. Since this first trial, many others corroborated trastuzumab benefit in survival outcomes among women with metastatic HER2+ BC.

Incorporation of new agents, as evidenced by the CLEOPATRA trial, in which pertuzumab, a humanized monoclonal antibody that binds to HER2 at a different epitope than that at which trastuzumab binds, was added to the standard docetaxel and trastuzumab combination, and lead to striking improvements in PFS and OS in a cohort of advanced HER2+ BC patients, reaching the median OS boundary of almost 5 years. [13–15]

Despite this robust clinical benefit, anti-HER therapy resistance, either de novo or acquired, is an important clinical challenge in the management of BC patients. Research has been dedicated to a better understanding of the molecular mechanisms involved of trastuzumab resistance. [16]

## MAIN RESISTANCE MECHANISMS PATHWAYS TO TRASTUZUMAB

### PIK3CA Pathway

#### Anti-HER2 Therapy Benefit and PIK3CA alterations

The PI3K/AKT/mTOR pathway is an important growth factor pathway and a key effector of HER2 signalling. HER2 phosphorylation may lead to pathway activation.[17] Constitutive activation of PI3K, either by PIK3CA mutation or PTEN loss, are associated with resistance to therapies targeting HER2, and possibly are able to identify a group of patients with poor prognosis after trastuzumab therapy. These alterations might result in continuous pathway signalling, despite HER2 blockage, priming a treatment escape mechanism. [18–20]

Many investigators evaluated trastuzumab benefit in patients enrolled in clinical trials in distinct disease scenarios, according to alterations in the PI3K pathway. Most of them failed to demonstrate a relationship between PIK3CA mutations and trastuzumab benefit. As an example, the FinHER adjuvant phase III trial genotyped 687 HER2+ BC patients. PIK3CA mutations were not statistically significantly associated with trastuzumab benefit, or survival outcomes. [21] Similarly, a recent metaanalysis also reached the conclusion that neither PTEN loss, nor PIK3CA mutation were associated with response rate of trastuzumab based neoadjuvant treatment. [22] Analysis of other trials also failed to demonstrate a relationship between PIK3CA or PTEN status and adjuvant trastuzumab benefit. [23, 24]

The EMILIA trial compared the effectiveness of TDM-1 versus lapatinib and capecitabine in patients previously treated with trastuzumab. Samples from patients were prospectively collected for PIK3CA mutation analysis. Patients in the lapatinib arm with PIK3CA mutations had worse outcomes than those with wild-type PIK3CA, but the presence of PIK3CA mutations had absolutely no effect on PFS or OS in patients treated with T-DM1, suggesting that this drug might be an attractive alternative for patients harbouring this alteration. [25]

The evidence described above is somehow contradictory to preclinical data. One important aspect to be taken into account is the fact that *in vitro* studies that initially identified PIK3CA mutation as a resistance factor for HER2-targeted treatment did not account for trastuzumab mediated antibody dependent cellular cytotoxicity (ADCC). Recognition of tumor cells opsonized by monoclonal antibodies, such as trastuzumab, is mediated through receptors expressed on effector cells, as well as monocytes, dendritic cells, and granulocytes. Upon recognition, these effectors induce tumor cell death. Dendritic cells capture the monoclonal antibody conjugated with tumor antigens released by dying cells in the form of immune complexes. Ultimately, these processed cells are presented to cytotoxic and helper T cells. As a consequence, both tumor-specific cytotoxic T cells and T-helper cells are activated, leading to tumor B-cell stimulation and expansion. The production of tumor-directed host antibodies is the final step in this important ADCC cytotoxic mechanism. [26] Also, trastuzumab enhances the ability of Natural Killer (NK) cells mediated ADCC, NK destruction of target cells through nonspecific mechanisms, ultimately leading to activation and expansion of tumor-specific cytotoxic T lymphocytes. [27] In fact, the importance of NK cell function is highlighted by the fact that there is a direct correlation between NK cell function and response to trastuzumab in metastatic HER2+ BC patients.[28] Preclinical models may not account for this, since many are conducted *in vitro* or in immunosuppressed models. Furthermore, trastuzumab is a human monoclonal antibody and therefore, unlikely to engender an immune reaction in a model system *in vitro*.

#### PIK3CA alterations as a prognostic marker

Survival outcomes of HER2+ BC patients might be influenced by PIK3CA status. Biomarker analysis from the CLEOPATRA trial demonstrated that PIK3CA mutation was associated with worse survival outcomes among patients with advanced HER2+ BC.[29] Nevertheless, the addition of pertuzumab benefited all patients, regardless of PIK3CA status.

Additionally, a group of researchers from Memorial Sloan-Kettering Cancer Center evaluated a group of 63 patients with HER2+ BC with disease recurrence after adjuvant trastuzumab treatment, or progressive metastatic disease on a trastuzumab-containing regimen.[20] All patients underwent biopsy of at least a single local or distant site to document progressive disease. PIK3CA and HER2 mutation status were analysed by Sequenon (genotyping DNA sequences), and HER2 and PTEN status were analysed by immunohistochemistry. Absent or significantly diminished PTEN expression was noted in 59%, and activating mutations in PIK3CA in 29% of cases evaluable for PTEN or PI3K mutation.

These authors also compared PTEN loss and PIK3CA mutation rate between 2 HER2+ BC cohorts of patients: one of them refractory to trastuzumab and the other unexposed to trastuzumab. They demonstrated that the combined rate of PTEN loss and PIK3CA mutation in the trastuzumab-refractory tumors was 71%, compared with 44% in the unexposed cohort, suggesting that trastuzumab exposure may lead to alterations in PIK3CA pathway.

Therefore, evidence supporting the hypothesis that PI3K alterations might lead to worse prognosis in HER2+ BC, as well as resistance to anti-HER2 therapy, is mostly based on small retrospective series. At the present time, the first (and still only) actionable genomic alteration in BC, is amplification of HER2, and anti-HER2 therapy is required in all disease scenarios where this alteration is observed.

#### Response to Neoadjuvant Chemotherapy combined with Anti-HER2 therapy according to PIK3CA status

Correlation between PI3K alterations and pCR achievement was extensively studied. Loibl and colleagues investigated whether the presence of a PIK3CA mutation affected the odds of achieving a pCR among patients enrolled in the GeparQuattro, GeparQuinto, and GeparSixto neoadjuvant clinical trials. [30] In these trials, HER2+ BC patients received either trastuzumab or lapatinib, or the combination plus anthracycline-taxane based chemotherapy.[31] The authors found that the pCR rate was lower among HER2+ women with at least one PIK3CA mutation in their tumor, compared with women without a PIK3CA mutation. In alignment with these results, investigators from the neoadjuvant TRYPHAENA study also found numerically lower pCR rates in patients with tumors carrying any PIK3CA mutation tested for, although this did not reach a statistically significant level, possibly due to small sample size. [32] Other neoadjuvant studies also found an association between PIK3CA mutation and lower rates of pCR, irrespectively of treatment arm.[33]

Of note, at the 2015 American Society of Clinical Oncology (ASCO) Meeting, investigators reported pCR rates according to PIK3CA status among HER2+ BC patients who received neoadjuvant trastuzumab, lapatinib, or both in addition to a taxane-based chemotherapy. Significantly lower rates of pCR were observed in PIK3CA mutant tumors after anti-HER2 treatment, namely 16.2% versus 29.6% (*p* < 0.001), for patients with and without a PIK3CA mutation, respectively. [34]

Table 1 summarizes differences in pCR rates according to PIK3CA mutation status.

**Table 1:** pCR Rates according to PIK3CA pathway alteration

%pCR	PIK3CA WT	PIK3CA MUT	
All treatments	34.5%	21.3%	Majewski et al., 2015
	29.6%	16.2%	Loibl et al., 2015
	32.8%	19.4%	Loibl et al., 2014
			

%pCR	PIK3CA WT	PIK3CA MUT	
Dual Anti-HER2 Therapy	55.8%	28.6%	Majewski et al., 2015
	39.1%	16.7%	Loibl et al., 2015
	37.1%	17.4%	Loibl et al., 2014

#### mTOR inhibitors combined with Anti-HER2 therapy

Therefore, much interest is focused on this important proliferative pathway that may contribute to trastuzumab resistance. Inhibition of mTOR has been shown to be an important therapeutic strategy in HER2+ BC. BOLERO-3 aimed to assess whether the addition of the mTOR inhibitor everolimus to trastuzumab might restore sensitivity to trastuzumab. Eligible patients for this trial had HER2+, trastuzumab-resistant, advanced BC who had previously received taxane therapy. Patients were randomized to receive daily everolimus (5 mg/day) plus weekly trastuzumab (2 mg/kg) and vinorelbine (25 mg/m2) or to placebo plus trastuzumab plus vinorelbine, in 3-week cycle. As a result, the addition of everolimus prolonged PFS from 5.78 months to 7.00 months (HR=0.78, *p*=0.0067).[35] In PFS subgroup analysis, most groups favoured the addition of everolimus, although women who had hormone receptor negative disease derived more benefit from adding the mTOR inhibitor to the standard therapy.

BOLERO-1 also focuses on the strategy of targeting the PI3K/AKT/mTOR pathway. In this trial, everolimus was added to paclitaxel and trastuzumab in patients with metastatic HER2+ BC patients who had not received previous trastuzumab or chemotherapy for advanced BC within 12 months of randomisation. Primary endpoint was PFS, and secondary endpoints included OS, response rate (RR), and clinical benefit rate. The results were presented at the 2014 San Antonio Breast Cancer Symposium (SABCS) and published in Lancet Oncology.[36] The study did not meet its primary objective in the full population, with comparable PFS among patients who received everolimus versus placebo. However, the authors found that in the hormone receptor (HR)-negative subpopulation, everolimus-treated patients achieved a median PFS of 20.27 months versus 13.08 months with placebo (HR = 0.66, P.0049).[37] Taken together, BOLERO-1 and 3 appear to demonstrate that the benefit of adding mTOR inhibitor to chemotherapy and trastuzumab in the metastatic disease setting is great in the HER2+ hormone receptor negative cohort.

Considering that mTOR inhibitors add a substantial amount of toxicity to therapy, biomarkers to identify patients who could derive more benefit from therapy are being studied. At the 2015 ASCO Meeting, researchers evaluated tumor tissue from patients recruited in BOLERO-1 and BOLERO-3. Exons of 282 cancer related genes were analyzed by next generation sequencing (NGS), and PTEN levels were evaluated by immunohistochemistry. Hyperactive PI3K pathway was defined as low PTEN levels or mutations in PIK3CA pathway. In both trials, patients with hyperactive PI3K derived more benefit from everolimus.[38]

Also, at 2013 ESMO (European Society of Medical Oncology) Conference, investigators presented data on patients from BOLERO-3 with low PTEN or high pS6 levels.[39] Those individuals were more likely to respond to the addition of everolimus to chemotherapy plus trastuzumab. In detail, patients with low PTEN levels achieved greater benefit with everolimus, with a median PFS absolute gain of about 18 weeks. Median PFS was 41.9 weeks with everolimus versus 23.1 weeks with placebo.

Contrasting with these results, biomarker analysis from BOLERO-2, which involved 3,230 exons of 182 oncogenes and tumor-suppressor genes that were sequenced using next-generation sequencing, on 309 tissue samples, demonstrated a positive treatment effect in favor of everolimus across the various key genetic marker subgroups.[40] In truth, a greater benefit from everolimus treatment was derived in patients with minimal genetic alterations in PIK3CA/PTEN/CCND1 or FGFR1/2 genes combined. Although BOLERO-2 [41] evaluated the benefit of adding everolimus in the treatment of a hormone receptor-positive, HER2-negative BC cohort, biomarker analysis evaluating alterations in PIK3CA pathway and mTOR inhibitors benefit, might provide some insights of everolimus benefit in other BC subtypes with specific somatic genomic alterations.

Other trials are currently being designed to specifically target the PI3K/AKT pathway in conjunction with HER2 itself, in order to hopefully achieve more durable and potent antitumor effects and overcome the resistant to treatment. These PI3K inhibitors are being introduced to the treatment of women with HER2+ BC in several stages of disease, from the neoadjuvant setting to the metastatic setting, after several lines of standard treatment.

As an example, the NeoPHOEBE study (NCT01816594), recruited women with HER2+ newly diagnosed early BC larger than 2cm. Patients were randomized to receive neoadjuvant weekly paclitaxel and Trastuzumab in combination with an oral PI3K inhibitor, BKM120, or placebo. The study has been completed, and was conducted in two separate cohorts (PIK3CA mutated and PI3K3CA wild-type) using a two-stage approach. NCT02152943 is evaluating everolimus, letrozole, and trastuzumab.

The combination of trastuzumab with a PI3K inhibitor was evaluated in a phase 1 clinical trial. Buparlisib plus trastuzumab was tested in a cohort of HER2+ metastatic BC patients resistant to trastuzumab-based therapy, with a manageable toxicity profile and preliminary evidence of clinical activity. Of note, inhibition of the PI3K/AKT/mTOR and RAS/MEK/ERK pathways was observed in paired tumor biopsies.[42] Pan PI3K inhibitors, such as pilaralisib (SAR245408), were tested in combination with trastuzumab and chemotherapy, with results showing objective evaluable responses.[43]

Recently, at ASCO 2015, an oral PI3Kα inhibitor, BYL719, was evaluated in combination with trastuzumab and a HER3 inhibitor, in a heavily pre treated HER2+ metastatic BC population (median number of previous therapy lines of 6). Clinical activity was observed in patients harbouring PIK3CA mutations. Nevertheless, gastrointestinal and metabolic toxicities were an important issue, warranting exploration of intermittent schedule regimens. [44]

The PIK3CA pathway may be targeted in other downstream points of the pathway, such as Akt. The Akt-inhibitor MK2206, was evaluated before surgery in patients with stage I, II or III BC with various combinations of ER, PR and HER2.

Many other trials are being performing with the important aim to target the PI3K/AKT/mTOR pathway in conjunction with HER2. Toxicity associated with PI3K/AKT/mTOR pathway inhibitors may be taken into account, as well as the added financial cost. Deaths attributable to everolimus were seen BOLERO-1, due to pneumonitis, pulmonary embolism, respiratory failure, pulmonary edema, pneumonia, and cardiorespiratory arrest. Management of adverse events related to these drugs, especially when combined with chemotherapy, is of extreme importance.

Therefore, the PI3K/AKT/mTOR pathway seems to play an important role in anti-HER2 therapy resistance. The optimal timing of targeting this pathway, either in the first line setting, or in a disease scenario where resistance is already established is yet to be determined. Also, the specific point of the pathway to be elected as the ideal target is still unclear. There are many options, ranging from mTOR-inhibitors to Akt-inhibitors and specific inhibitors of different isoforms of PI3K. Additionally, a balance between cost and side effects is of extreme importance in moving forward this strategy to clinical practice.

### p95HER2

The aminoterminal-truncated form of the HER2 receptor, that undergoes a proteolytic cleavage generating a truncated fragment, is denominated p95HER2 and is constitutively active. Also, p95HER2 fragments may arise through translation of the mRNA encoding HER2 from internal initiation codons.[45] It has kinase activity, but lacks the extracellular domain and the binding site of trastuzumab, enabling activated signalling despite the presence of trastuzumab.[46]

The presence of p95HER2 has been associated with worse survival outcomes in patients with localized HER2+ BC. Primary breast tumor tissues were evaluated by Western blot analysis for HER-2 protein forms, including p185HER-2 and p95HER-2. Authors found that a high level of p95HER-2 in primary tumor tissue correlated with reduced 5-year disease-free survival (DFS) (HR 2.55; 95% IC, 2.13-8.01; P < 0.0001). Differences in DFS were quite impressive between the two groups, namely 139 versus 32 months, for low and high levels of p95HER2, respectively.[47]

Despite being an independent prognostic factor in breast cancer, defining a group of patients with HER2+ disease with significantly worse survival outcomes, p95HER2 is also a marker of resistance to trastuzumab. Previous studies demonstrated that metastatic BC patients overexpressing p95HER2 had considerably lower response rates to trastuzumab compared with patients expressing full-length HER2.[46] In this trial, Scaltriti et al. also evaluated the effect of lapatinib in a p95HER2 preclinical model of p95HER2-positive MCF-7 cells. Lapatinib, a small molecule that inhibits both HER2 and EGFR kinases, successfully inhibited p95HER2 phosphorylation, reducing downstream phosphorylation of Akt and MAPK, with cell growth inhibition.[46]

Other studies reported a correlation between elevated p95 expression and poor outcomes in response to trastuzumab treatment.[48, 49]

Interestingly, recent studies showed that tumors expressing the most active p95HER2 fragment, the so-called p95HER2/611CTF, indeed do respond to trastuzumab in combination with chemotherapy. The 611CTF fragment is highly oncogenic, because it spontaneously homodimerizes into a constitutively active form.[50] *In vitro* experiments observed that chemotherapy sensitizes p95HER2/611CTF-positive patient-derived xenografts to trastuzumab, which may be related to HER2 stabilization induced by chemotherapy.[51] Another analysis on the p95HER2/611CTF isoform on samples from the NeoALTTO trial also demonstrated better responses to trastuzumab in combination with paclitaxel in tumors that expressed high levels of this isoform.52

**Figure 1:**
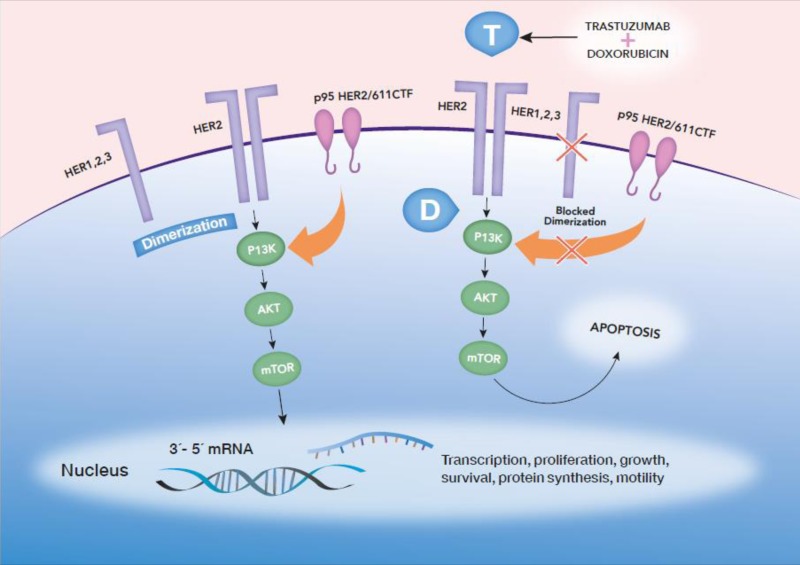
HER2 dimerizes with other partners from the HER2 family, activating intracellular proliferative pathways Also, p95HER2/611CTF has intrinsic kinase activity, lacking the extracellular domain and the binding site of trastuzumab (T), enabling activated signaling, despite the presence of trastuzumab. Upon activation of proliferative pathways, such as PI3K/AKT/mTOR, the cell undergoes proliferation. Trastuzumab blocks HER2 dimerization and phosphorylation, but p95HER2/611CTF might still phosphorylate and enable cancer cell proliferation. In contrast, treatment with doxorubicin (D) and trastuzumab induces apoptosis more efficiently in p95HER2/611CTF-positive cells and, destabilizing phospho-HER2 and stabilizing HER2 in p95HER2/611CTF-positive cells. Therefore, cells are sensitized to trastuzumab and suffer apoptosis through trastuzumab therapy.

Larger studies are highly awaited for confirmation of p95HER2 as a biomarker of resistance or sensitivity to anti-HER2 therapy, enabling development of strategies to target this form of HER2. Evidence is accumulating indicating that trastuzumab alone is not effective in patients who have expression of p95HER2 isoforms. Figure 1 illustrates HER2 and p95HER2, along with their interactions with chemotherapy and trastuzumab, as well as proliferative molecular pathways.

Chemotherapy, in conjunction with trastuzumab, is essential to achieve response to therapy. Larger and well-controlled studies, using pre-defined clinical cutoffs for p95 expression and also blinded analyses are highly awaited.

### Insulin-like growth factor-I receptor (IGF-IR)

Insulin-like growth factor-I receptor (IGF-IR) is another pathway implicated in trastuzumab resistance.[53] This receptor tyrosine kinase plays an important role in tumor cell growth and survival. Upon ligand stimulation, IGF-IR generates signaling through the Ras/mitogen-activated protein kinase and phosphatidylinositol 3-kinase/AKT pathways, stimulating cell growth, proliferation and apoptosis inhibition.[54]

Important data demonstrated that interaction between HER2 and IGFR-IR occurs in trastuzumab-resistant cells and that inhibition of IGF-IR tyrosine kinase activity may lead to decreased HER-2 phosphorylation with consequent restoration of trastuzumab sensitivity. This important cross talk between HER-2 and IGF-IR could potentially lead to clinical use of IGF-IR-targeted agents to block this receptor.

*In vitro* studies demonstrated that inhibition of HER2 signaling using trastuzumab, and inhibition of IGF-IR signaling using a dominant negative construct produced synergistic growth inhibition of HER2-overexpressing breast cancer cells.[55, 56]

Of note, in experiments in which *in vitro* inhibition of IGF1R expression was made through small receptor tyrosine kinase inhibitors, response to trastuzumab was substantially improved, suggesting an interaction between HER2 and IGFR. [57]

Future clinical trials of HER2 and IGF1R-inhibitors are awaited to corroborate these preclinical findings. Analysis of potential predictive biomarkers to identify tumors that may achieve maximum benefit from this approach would be of great interest.

### *MET* Aberrations

c-MET is a receptor tyrosine kinase (RTK) encoded by the MET proto-oncogene that is largely expressed in epithelial/endothelial cells. [58] Upon binding with the hepatocyte growth factor (HGF), the c-MET receptor dimerizes, autophosphorylates and activates downstream pathways, including mitogen-activated protein kinase (MAPK), phosphatidylinositol 3-kinase (PI3K) and signal transducer and activator of transcription.[59, 60]

Overexpression of c-MET has been shown to contribute to the development of the invasive phenotype during BC progression. Poorly differentiated and invasive cell lines may express high levels of the receptor and ligation by HGF is associated with increased motility and invasiness.[61]

Previous trials have shown that overexpression of c-MET in BC primary tumors is strongly associated with worse disease-free survival compared to tumors without c-MET overexpression.[62, 63]

MET expression might be associated with resistance to treatment in BC patients, even in a cohort of individuals co expressing ERBB2, in which a highly effective treatment, such as trastuzumab, is largely available.[64]

In fact, overexpression c-Met has emerged as a potential contributor to trastuzumab resistance and has been shown to be highly elevated in HER2-positive breast cancer cell lines and in 25% of HER2-positive breast cancer patients tissues.[62, 65] Loss of Met function is implicated in the development of trastuzumab response.[65]

Cell line experiments demonstrated that foretinib, a multikinase MET inhibitor, combined with erlotinib or lapatinib, was capable of cell growth inhibition of MET-amplified or overexpressing cell lines.[66] Additionally, in the presence of HGF, these cell lines demonstrated reduced sensitivity to lapatinib, which may indicate that MET activation can decrease the effectiveness of HER1/2 inhibitors.

Many therapeutic strategies to target Met are currently under clinical development, including tyrosine kinase inhibitors and monoclonal antibodies, which could potentially be associated with anti-HER2 therapy in patients with BC overexpressing HER2 to optimize treatment and avoid tumor resistance. As an example, cabozantinib, a small molecule inhibitor of the tyrosine kinases c-Met and VEGFR2, is currently being evaluated in combination with trastuzumab, in HER2+ BC patients who have brain metastasis (NCT02260531).

### Src

The proto-oncogene Src encodes for the non-receptor protein tyrosine kinase Src, which is involved in many cellular events, mediating cell proliferation and survival. [67]

Src has extensive interaction with transmembrane receptor tyrosine kinases (RTKs) at the cell membrane, such as HER1 and HER2. Src activation confers resistance to trastuzumab, both acquired and *de novo*. In fact, data points to the fact that Src activation by itself is sufficient to confer trastuzumab resistance.[68]

The relationship between SRC activation and patient response to trastuzumab-based therapies was previously studied in a cohort of 57 patients who were evaluated for SRC activation in primary breast tumors. As featured, patients with high amounts of phosphorylated Src (pSrc) in tumors demonstrated a lower clinical response rate and a higher progressive disease rate after trastuzumab therapy. Additionally, the OS of patients with high pSrc tumors (median 34.2 months) was significantly lower than that of patients with low pSrc tumors (median 57.9 months).[68] *In vitro* targeting Src universally sensitized trastuzumab-resistant cells to trastuzumab treatment, and was able to suppress tumor growth in multiple preclinical resistance models. Other studies also demonstrated that Src activation is involved with trastuzumab mechanisms of resistance, and indicates poor prognosis in patients with HER2+ BC.[69]

Resistance to lapatinib was also demonstrated in Src activated cell lines. Saracatinib, a small molecule inhibitor of Src, was combined with lapatinib, and significantly prolonged survival of xenografted mice compared with saracatinib alone.[70]

Therefore, blocking Src interaction with HER2 is a promising strategy that might impact the management of HER2+ BC patients.

### Immune Response and trastuzumab sensitivity

Trastuzumab mechanism of action is dependent on the host immune system. As a consequence, trastuzumab is only partially effective in mice defective for the receptor of immunoglobulins (gamma receptor knockout mice). Innate and adaptive immune mechanisms are emerging as key players in modulation of the effects of HER2-targeted drugs.[71]

Therefore, sensitivity to anti-HER2 therapy depends on a well functioning host immune system. Loi et al. evaluated tumor PIK3CA mutations, lymphocyte infiltration, and RFS in an early HER2+ BC cohort of patients enrolled on the Finher Trial. [72, 73] In this analysis, the presence of tumor lymphocyte infiltration (TIL) was associated with prediction of trastuzumab response.[73,74]

In the neoadjuvant setting, signals of immune system activation were associated with response to pertuzumab and trastuzumab in the Neosphere study. In this analysis, baseline core biopsies were collected from 387 out of 417 patients and evaluated by gene expression profiling. Patients identified as having low expression of PD-L1 had lower pathologic complete response rates (pCR). In contrast, individuals with high expression of STAT1 and IFNG, achieved high pCR rates, irrespective of estrogen receptor status.[75]

Tumor cells display genetic and epigenetic changes that provide plenty of tumor-associated antigens, which could potentially elicit host immune system recognition and tumor cell destruction. Avoidance of this host intrinsic defense mechanism is fundamental for tumor progression. As a consequence, immune-checkpoint proteins are frequently deregulated by tumors.[76] Cytotoxic T-lymphocyte antigen 4 (CTLA-4), which is a negative regulator of T cell activation, and PD-1 (programmed cell death protein 1), which is an immune checkpoint that limits the activity of T cells in peripheral tissues, are immune checkpoint proteins that are currently being targeted in a variety of solid tumors, such as melanoma, lung, renal and also breast cancer.

Among one of the first reports that recognized the importance of this rapidly evolving field in BC was presented at the 2013 SABCS.[77] Breast tumor samples from 156 patients with HER2-positive BC enrolled in the GeparQuattro trial were evaluated. In this cohort of patients, for every 10 percent increase in the levels of tumor-infiltrating lymphocytes there was a 16 percent increase in the number of patients who had a pCR. Furthermore, authors investigated samples from patients recruited in the FinHer trial, and found that high levels of CTLA-4 and PD-1 correlated with a survival benefit after trastuzumab treatment in a mouse model. Besides, treatment of mice with trastuzumab and either an agent that blocks PD-1 or PD-L1, resulted in greater tumor regression compared with trastuzumab alone, suggesting an important role of checkpoint-inhibitors in HER2+ BC.

Recently, Perez et al. defined a group with enriched immune functions in patients enrolled in the N9831 trastuzumab adjuvant trial, based on the expression of 87 genes.[78] Using genomic technology, the authors identified significantly enriched biological processes associated with increased RFS that were linked to immune functions. This group was denominated immune group and experienced more favorable outcomes when treated with trastuzumab. Of note, patients who did not exhibit immune function enrichment and were treated with trastuzumab did not experience better RFS compared to patients with immune function enrichment who were treated with chemotherapy alone (HR=0.93, p=0.72). Therefore, a state of immunological function appears to be associated with HER2-targeted therapy efficacy and warrants further evaluation in future trials.

Analysis of immune markers in HER2+ BC indicates that host immune system integrity is extremely important to trastuzumab response. Future clinical trials exploring patient immune system, with the ultimate goal of maximizing immune response, might result in great improvement in trastuzumab benefit and sensitivity.

Table 2 summarizes the main resistance mechanisms to trastuzumab therapy, correlating with associated strategies to overcome it.

**Table 2:** IGFR Insulin growth factor receptor; TIL tumor infiltrating lymphocytes

Mechanisms of Trastuzumab resistance and strategies to target it		
Resistance Mechanism		Strategy to Overcome resistance Anti-HER2 Therapy plus
PIK3CA Alterations	PIK3CA Mutation PIK3CA Copy Number gain PIK3CA Amplification PIK3CA Amplification PIK3CA Increased Expression PTEN loss	Pan PI3K Inhibitor Specific PIK3CA Inhibitors AKT Inhibitors mTOR Inhibitors
High Levels of p95HER2		Chemotherapy Lapatinib
IGFR1 tyrosine kinase receptor activation		IGF1R monoclonal antibodies or tyrosine kinase inhibitors
MET Aberrations	MET Amplification MET Mutation	MET inhibitors
Low Immune Response	Low TIL levels Lack of immune genes expression	Immune checkpoint inhibitors

## MAIN RESISTANCE MECHANISMS PATHWAYS TO OTHER ANTI-HER2 THERAPIES

### Lapatinib and Other Tyrosine Kinase Inhibitors (TKIs)

Lapatinib is a synthetic, orally active TKI that reversibly blocks phosphorylation of the epidermal growth factor receptor (EGFR), HER2, Erk-1 and-2 and AKT kinases [79]. Other TKIs that irreversibly block phosphorylation of these receptors include neratinib, afatinib (BIBW2992), CUDC-101, and canertinib (CI-1033), among others. Irreversible TKIs, such as neratinib, could have the theoretical advantage to potentially revert first-generation HER receptor tyrosine kinase inhibitors resistance [80].

Lapatinib in combination with capecitabine is FDA-approved for the treatment of HER2+ metastatic BC that have received prior therapy with trastuzumab [7]. Also, it is approved in combination with letrozole for the treatment of postmenopausal women with HER2+ and hormone receptor-positive metastatic BC, for whom hormonal therapy is indicated [81].

Combining trastuzumab with lapatinib is an attractive strategy, since both intracellular and extracellular HER2 domains would be targeted. Indeed, *in vitro* synergistic interaction was demonstrated between those two drugs [82]. Dual anti-HER2 blockade with lapatinib and trastuzumab improved PFS and clinical benefit rate in women with metastatic HER2+ BC [83], and also improved pathologic complete response rate in locally advanced HER2+ BC patients who received neoadjuvant chemotherapy [84, 85]. Nevertheless, lapatinib in combination with trastuzumab did not improve survival outcomes in the adjuvant setting [86].

Unfortunately, acquired resistance to lapatinib often develops in patients who initially responded to therapy. Chronic exposure to lapatinib may convert HER2-over-expressing BC cells that are initially sensitive to lapatinib-induced apoptosis to resistant cells [87]. Importantly, in cell lines that display overexpression of both estrogen receptor (ER) and HER2, FOXO3a and ER-regulated gene products are actually induced by lapatinib. Also, lapatinib modulates the expression of proteins that promote transcription of ER-regulated genes. Therefore, it is hypothesized that regulation of cell survival switches from HER2 to ER during the development of acquired resistance to lapatinib [88]. In cell lines that are ER-, resistance to lapatinib appears to be an ER-independent process.

Small molecule TKIs are being studied in combination with drugs that target the PIK3CA/AKT pathway, which was previously discussed as being implicated in trastuzumab resistance. PI3K inhibitors in monotherapy might display modest clinical activity, and a potential resistance mechanism frequently involves increased expression and phosphorylation of the HER family of receptors, particularly HER2 and HER3 [89, 90]. Therapeutic combinations to target both pathways could prevent or delay the development of resistance. Actually, combining a PI3K inhibitor with a small molecule inhibitor of EGFR/HER2/HER3 signaling was shown to have synergistic growth inhibition in breast cancer cell lines *in vitro*, suggesting that this is a strategy that might be a possible treatment option to be tested in future clinical trials [91].

Another potential mechanism to lapatinib resistance is activation of AXL, which is a membrane-bound receptor tyrosine kinase [92]. In a lapatinib-resistant BC cell clone model, AXL expression was increased and treatment with GSK1363089, a potent inhibitor of AXL, MET, and VEGFR, restored drug sensitivity [93]. Also, phosphorylation of AXL is reported to be associated with HER2 signaling, leading to downstream AKT activation, and generating activation of the AKT/mTOR intracellular proliferative pathway [94].

### Pertuzumab

This humanized monoclonal antibody that binds to HER2 is an effective inhibitor of EGFR/HER2 driven signaling pathway. Compared to trastuzumab, much less is known about development of resistance to this targeted agent. Of note, in a tamoxifen-resistant BC cell line, pertuzumab promoted rapid formation of HER3/EGFR heterodimers, with subsequent phosphorylation of AKT and ERK1/2 [95]. In this cell line model, the rapid formation of HER3/EGFR heterodimers provided a mechanism whereby epidermal growth factor stimulation could overcome the growth inhibitory effect of this agent. Moreover, HER3/EGFR heterodimer levels were increased in tamoxifen/pertuzumab resistant cell lines, further suggesting that pertuzumab-induced EGFR/HER3 heterodimerization might play a role in the rapid acquisition of resistance to this agent in tamoxifen-resistant cells.

In ovarian cancer, microRNA-150 (miR-150) expression was induced by pertuzumab in a cell line model, and suppression of miRNA-150 resulted in decreased drug sensitivity to pertuzumab and cell apoptosis [96]. miRNAs are small noncoding, single-stranded RNAs that regulate several biological processes, controlling gene expression at a posttranscriptional level [97]. miRNA-150 was found to be a negative PI3K-AKT pathway regulator. Also, an AKT inhibitor, was used to treat pertuzumab resistant cancer cells, and was capable of achieving anti tumor effects [96]. Although this model was performed in an ovarian cancer cell line, this might provide some insights to breast cancer as well.

The development of resistance to anti-HER2 therapies seems complex, and is probably associated with multiple mechanisms. A generated transgenic mouse model of HER2+, PIK3CA mutant BC acquired resistance to trastuzumab, pertuzumab in combination with the pan-PI3K inhibitor BKM120 after treatment exposure. Initially, tumor regression occurred after 6 weeks of therapy. Nevertheless, within two months, all tumors relapsed. Of note, p95 HER2, which was not detected in untreated tumors, was expressed in resistant clones, and HER2 expression was significantly reduced [98]. This data suggests that a wide range of heterogeneity of mechanisms of acquired resistance may occur in different HER2+/PIK3CA-mutant BC patients.

### T-DM1

Trastuzumab emtansine (T-DM1) is an antibody-drug conjugate that is active in second line, as well as later lines in the treatment of advanced HER2+ BC that has progressed on trastuzumab therapy [99, 100]. Although most patients will develop resistance to this agent, the mechanisms associated with this phenomenon are incompletely understood.

Primary resistance to T-DM1 may be relatively infrequent, particularly in patients who have no prior exposure to trastuzumab. The most common scenario is found in patients who initially responded, but eventually ceased to respond, despite continued treatment with T-DM1. It is important to know that DM1 and its metabolites need to accumulate in cell cytoplasm to reach a concentration that exceeds the threshold needed to evoke cell death [101]. Consequently, factors that affect the ability of the drug to reach a satisfactory intracellular level might be implicated in lack of clinical efficacy. Low tumor HER2 expression, poor internalization of the HER2-T-DM1complexes, defective intracellular and endosomal trafficking of the HER2-T-DM1 complex and defective lysosomal degradation of T-DM1 are all associated with low intracellular DM1 levels [102]. Also, drug efflux pumps might be implicated in resistance to therapy and expression of multi-drug resistance transporters may play a role in T-DM1 resistance.

Another important resistance mechanism of T-DM1 is mediated by neuregulin b1 (NRG) [103], the ligand protein of HER3, encoded by the NRG1 gene [104]. The presence of NRG can suppress the cytotoxic activity of T-DM1 in BC cell lines. This effect was reversed when cells were treated with pertuzumab. NRG can trigger the formation of HER2-HER3 heterodimers, a potent activating mechanism of the PI3K pathway, which was previously described in this review as a proliferative pathway involved with anti-HER2 therapy resistance [105].

Other HER2-targeting antibody-drug conjugate (ADC) are currently under development, such as SYD985. In the ongoing phase 1 clinical trial, safety and efficacy of SYD985 have been evaluated in European Oncology Centers, where patients with locally advanced or metastatic solid tumors of any origin were treated with SYD985. Of note, objective responses were seen in metastatic HER2+ BC patients who were refractory to previous HER2-targeted treatments, including T-DM1 [106].

## CONCLUSIONS

Resistance to trastuzumab and other anti-HER2 therapies is an event that may occur during the course of therapy or *de novo*. In either case, this is a potentially life-threatening event, which may lead to rapidly progressive disease, or can result in important quality of life impairment for patients, due to disease related symptoms. A better knowledge of these mechanisms is of extreme importance for the development of effective strategies to overcome resistance.

In developing more effective and durable treatment strategies, multitargeted therapy should be considered. The recognition of specific molecular predictors of response to emerging therapies will allow a more personalized approach to the treatment of HER2-amplified BC.

Enrolling patients into clinical trials, with the purpose of understand and target the molecular mechanisms involved in HER2 therapy resistance is of crucial importance. Laboratory, pre clinical and clinical findings, such as seen at the BOLERO-1 and 3 trials, are very promising in this field, but have not yet been incorporated in clinical practice. Also, a better characterization between the relationship of tumor inflammatory infiltrate or molecular inflammatory signature, and trastuzumab benefit is warranted, before its incorporation in clinical practice.

Nowadays, the focus on HER2 expression/amplification status alone is not a realistic approach to understand the underlying mechanisms of disease progression and resistance. Improvement of patient outcomes in this disease setting will only be clinically meaningful if resistance pathways involved in disease progression are better recognized, understood and targeted.
